# Supplementary material for: Synergy between Phage Sb-1 and Oxacillin against Methicillin-Resistant Staphylococcus aureus
Source: Antibiotics (Basel). 2021 Jul 13;10(7):849. doi: 10.3390/antibiotics10070849 (PMC8300854; doi:10.3390/antibiotics10070849)
Supplement: Supplementary file 1 [file antibiotics-10-00849-s001.zip › FigureS1.pdf]

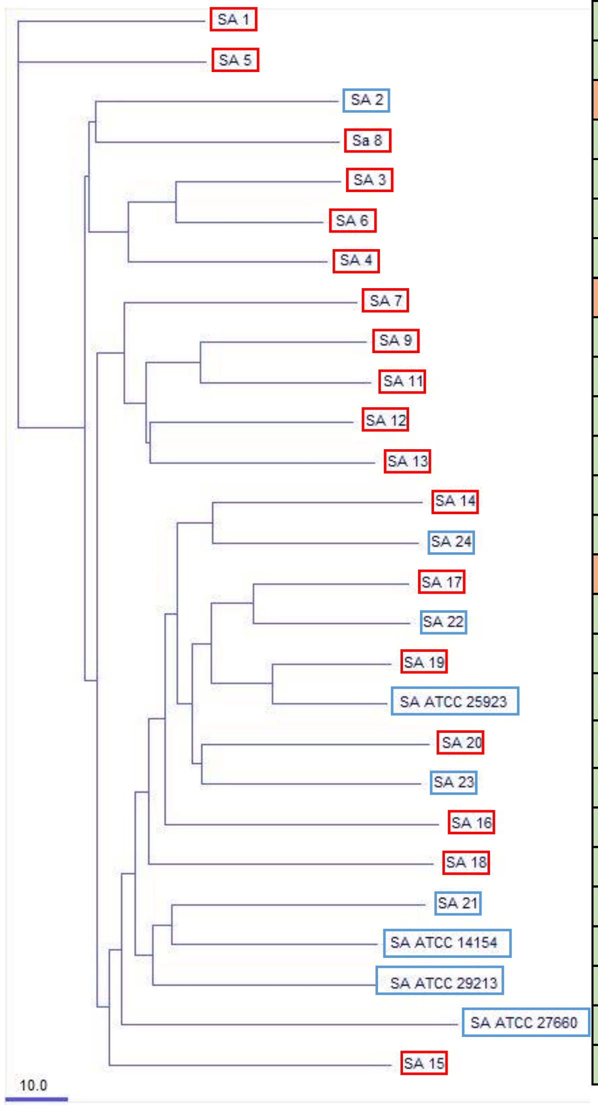

Phage lyis	Oxa	Levo	Genta	Clinda	Co-tri	Tetra	Erythro	Mupi
S	R	R	S	R	S	S	R	S
S	R	R	S	R	S	S	R	I
R	S	S	S	S	S	S	S	S
S	R	R	S	R	S	S	R	S
S	R	R	S	R	S	S	R	S
S	R	R	S	S	S	S	S	S
S	R	R	S	S	S	I	S	S
R	R	R	R	S	R	S	S	S
S	R	R	S	S	S	S	S	S
S	R	R	S	R	S	S	R	S
S	R	R	S	S	S	S	S	S
S	R	R	S	S	S	I	S	S
S	R	R	S	R	S	S	R	S
S	S	S	S	S	S	R	S	S
R	R	I	S	R	S	R	R	S
S	S	S	S	S	S	S	S	S
S	R	R	S	S	S	S	S	S
S	n.d.	n.d.	n.d.	n.d.	n.d.	n.d.	n.d.	n.d.
S	R	R	S	R	S	S	R	S
S	S	S	S	S	S	S	S	S
S	R	R	S	R	S	S	R	S
S	R	R	S	R	S	S	R	S
S	S	R	S	S	S	S	S	S
S	n.d.	n.d.	n.d.	n.d.	n.d.	n.d.	n.d.	n.d.
S	n.d.	n.d.	n.d.	n.d.	n.d.	n.d.	n.d.	n.d.
S	n.d.	n.d.	n.d.	n.d.	n.d.	n.d.	n.d.	n.d.
S	R	R	S	S	S	S	S	S

Fig. S1 Neighbor-joining dendrogram resulting from ERIC-PCR for 27 *S. aureus* isolates: 18 clinical MRSA (red rectangle), 5 clinical MSSA, and four ATCC reference MSSA strains (blue rectangle) in conjunction to the susceptibility to phage Sb-1 along with antibiotic resistance profiles. Oxa: Oxacillin; Levo: Levofloxacin; Genta: Gentamicin; Clinda: Clindamycin; Co-tri: Trimethoprim/sulfamethoxazole; Tetra: Tetracycline; Erythro: Erythromycin; Mupi: Mupirocin; R = resistant, I = intermediate, S = sensitive, n.d.: not determined;
