# Supplementary material for: Synergy between Phage Sb-1 and Oxacillin against Methicillin-Resistant Staphylococcus aureus
Source: Antibiotics (Basel). 2021 Jul 13;10(7):849. doi: 10.3390/antibiotics10070849 (PMC8300854; doi:10.3390/antibiotics10070849)
Supplement: Supplementary file 1 [file antibiotics-10-00849-s001.zip › FigureS2.pdf]

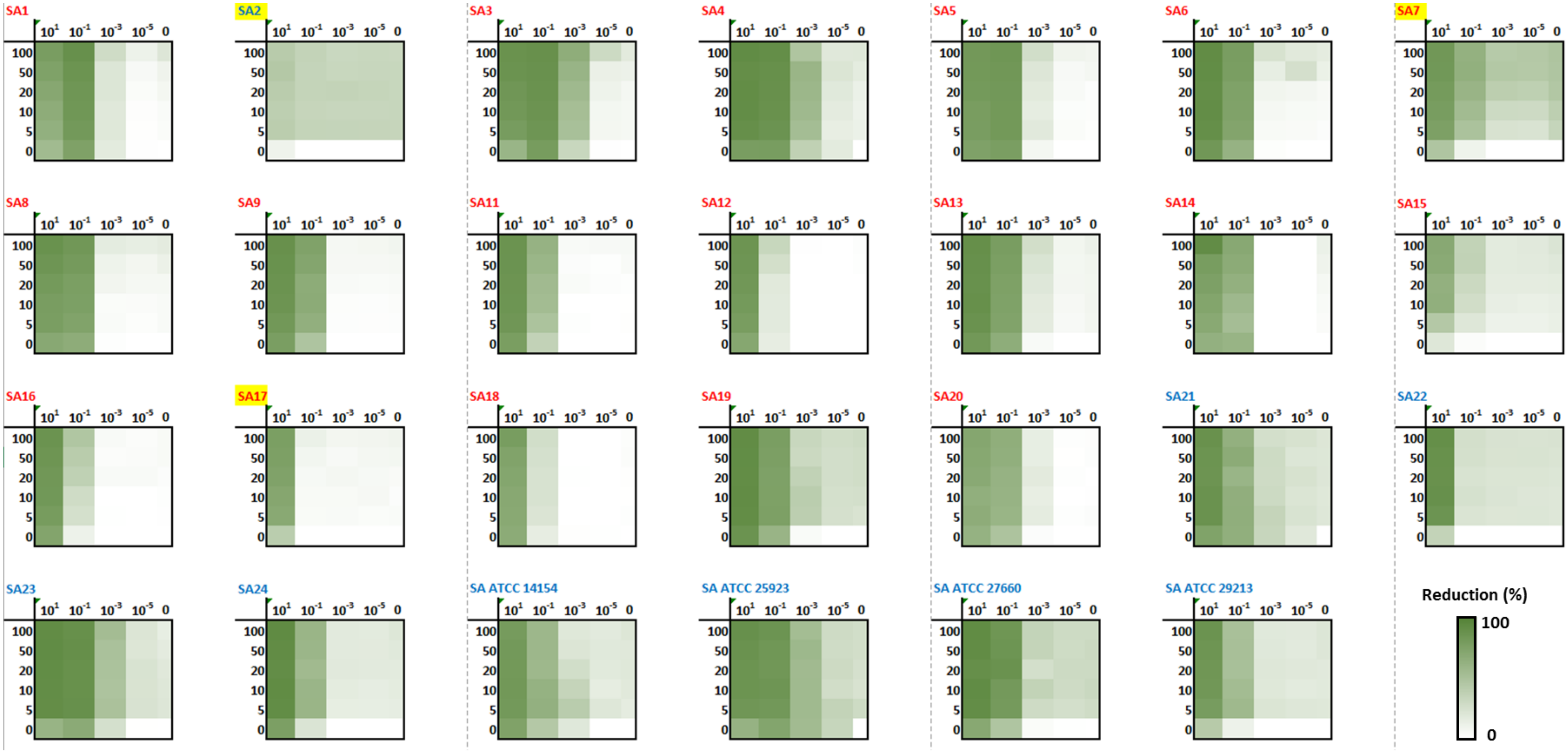

Figure S2: Heatmap displaying the level of bacterial reduction based on combination assays with varying dosages of phage Sb-1 (MOI 0, 10^{-5}, 10^{-3}, 10^{-1}, 10, horizontal axis) and oxacillin (0, 5, 10, 20, 50, and 100 µg/ml, vertical axis) against 27 S. aureus isolates SA1 to SA24 (SA10 not included) plus four ATCC strains. Red: MRSA, Blue: MSSA, Yellow: Phage-resistant isolates (all other isolates are phage sensitive)
