# Supplementary material for: Synergy between Phage Sb-1 and Oxacillin against Methicillin-Resistant Staphylococcus aureus
Source: Antibiotics (Basel). 2021 Jul 13;10(7):849. doi: 10.3390/antibiotics10070849 (PMC8300854; doi:10.3390/antibiotics10070849)
Supplement: Supplementary file 1 [file antibiotics-10-00849-s001.zip › FigureS3.pdf]

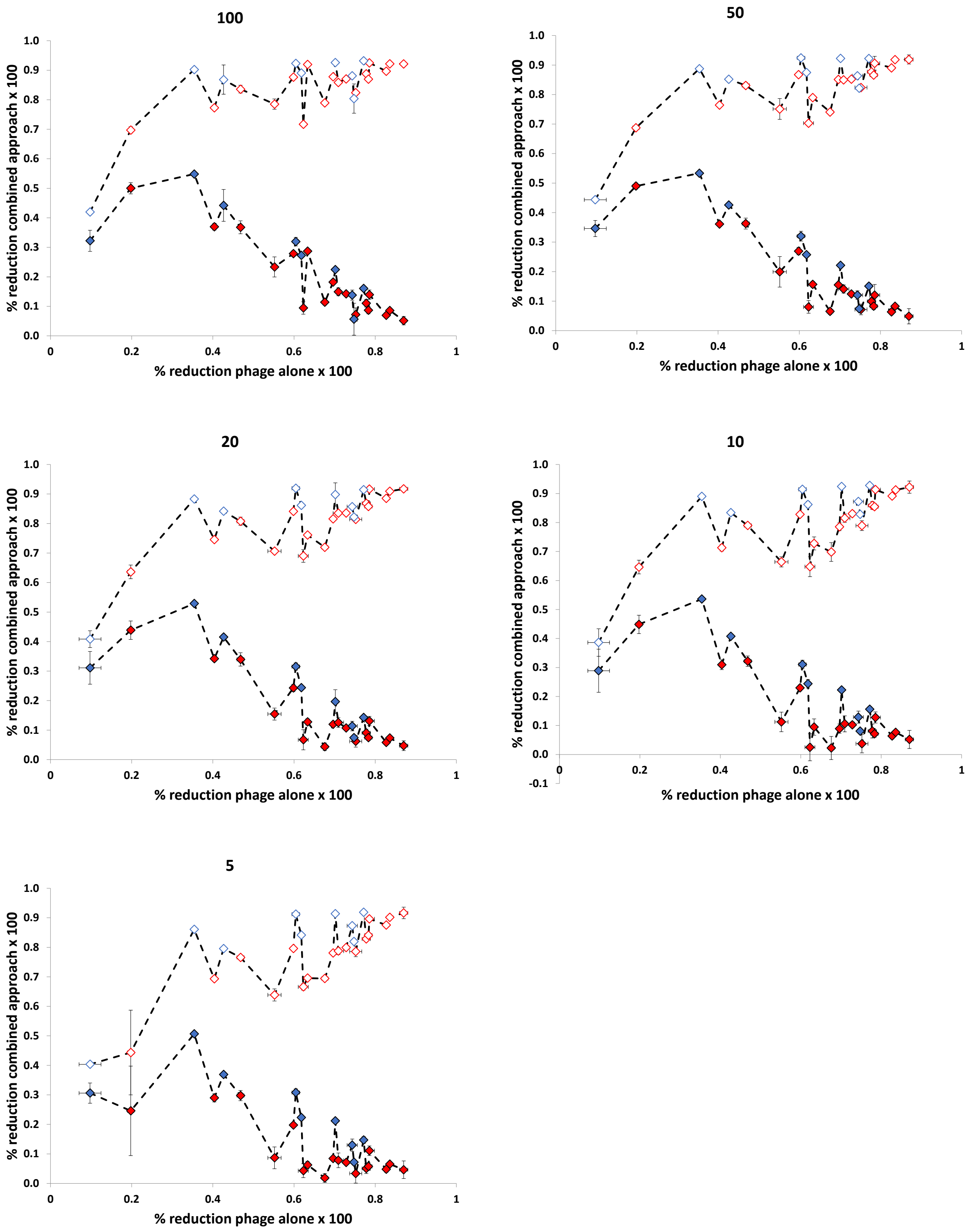

Supplemental Figure S3: Association between the antibacterial efficacy of the phage Sb-1 alone (MOI 10) and the combination approach using Sb-1 and oxacillin together. Each of the 27 *S. aureus* isolate tested in this study is represented by one filled diamond and one overlying empty diamond (red: MRSA, blue MSSA). The horizontal bar depicts the percent reduction with the phage-alone approach. The vertical axis depicts the additional reduction achieved with the combined approach (filled diamonds) and the total bacterial reduction of

the combined approach (empty diamonds). The numbers 5, 10, 20, 50, and 100 on top of each diagram indicate the oxacillin concentrations (µg/ml). The diagrams indicate, that for strains, where the phage alone approach is already highly efficient, the combination approach contributes only little. Conversely, when the level of bacterial reduction by the phage alone lies somewhere between 30 to 50% only, the combination approach accounts substantially for the additional bacterial reduction. This shows, that co-addition of oxacillin is particular meaningful for *S. aureus* strains (including MRSA), that are not so well suppressed by the phage alone.
