# Supplementary material for: Synergy between Phage Sb-1 and Oxacillin against Methicillin-Resistant Staphylococcus aureus
Source: Antibiotics (Basel). 2021 Jul 13;10(7):849. doi: 10.3390/antibiotics10070849 (PMC8300854; doi:10.3390/antibiotics10070849)
Supplement: Supplementary file 1 [file antibiotics-10-00849-s001.zip › Supplemental_Information_Methods.pdf]

Synergy between phage Sb-1 and oxacillin against Methicillin-resistant *Staphylococus aureus*

Kevin Simon[1], Wolfgang Pier[1], Alex Krüttgen[2], Hans-Peter Horz[1]

[1]Institute of Medical Microbiology, RWTH Aachen University Hospital, 52074 Aachen, Germany

[2]Laboratory Diagnostic Center, RWTH Aachen University Hospital, 52074 Aachen, Germany;

Supplemental Information Methods

Phage storage and time-kill assays

For short-term storage (e.g. several weeks) phage lysates were stored at 4 °C. For long-term storage phages were mixed with glycerol (20% (v/v)) in equal parts and stored in CryoPure Tubes (Sarstedt, Nuembrecht, Germany) at −196 °C in liquid nitrogen.

For the infection assays, 10 ml of 2x LB were inoculated with 100 µl of the host strain and incubated overnight at 37°C and 200 rpm. Subsequently, 5 ml of this culture were again mixed with 5 ml 2x LB and shaken at room-temperature for another hour to reach bacterial concentration of approximately $5x10^8$ CFU/ml. Phage-lysates were diluted by ten-fold serial dilution. 100 µl of phage-lysates were inoculated with 98 µl of bacterial strain and 2 µl of the selected antibiotic on a 96-well microtiter plate. For controls, 98 µl of the host strain or LB medium were mixed with 2 µl RNase-free water and 100 µl PBS. After sealing the microtiter plate with an adhesive tape, a hole was made in every well to supply the bacteria with oxygen throughout the experiment. The microtiter plate was then placed into the microplate reader SpectraMax i3 (Molecular Devices, Sunnyvale, United States of America) where the OD_{590} at 37°C was measured every 20 minutes over 16h. The OD_{590} data were used for calculating the area under the curve (AUC) via numerical integration with the formula $\sum_{i=0}^{48} \frac{f(i*\Delta t)+f(i+1*\Delta t)}{2} * \Delta t$, with $\Delta t = 20\ min\ = 0.33\ h, and f(i * \Delta t)$ representing the OD_{590}-values measured every 20 min for 16h.[1] Bacterial reduction in % was calculated as AUC of the growth control (no treatment) minus AUC of the treatment divided by AUC of the growth control x 100.

[1]Jansen M, Wahida A, Latz S, *et al.* Enhanced antibacterial effect of the novel T4-like bacteriophage KARL-1 in combination with antibiotics against multi-drug resistant Acinetobacter baumannii. *Sci Rep* 2018; **8**. Available at: http://www.nature.com/articles/s41598-018-32344-y. Accessed October 1, 2018.
